# Incentivised physical activity intervention promoting daily steps among university employees in the workplace through a team-based competition

**DOI:** 10.3389/fpubh.2023.1121936

**Published:** 2024-01-22

**Authors:** Ayazullah Safi, Sanjoy Deb, Adam Kelly, Matthew Cole, Natalie Walker, Mohammed Gulrez Zariwala

**Affiliations:** ^1^Department of Public Health, Centre for Life and Sport Science (C-LaSS) at Birmingham City University, Birmingham, United Kingdom; ^2^Cambridge Centre of Sport and Exercise Science, Anglia Ruskin University, Cambridge, United Kingdom; ^3^Sport and Exercise Science, Centre for Life and Sport Science (C-LaSS) at Birmingham City University, Birmingham, United Kingdom; ^4^Hartpury University, Gloucestershire, United Kingdom; ^5^School of Life Sciences, Coventry University, Coventry, United Kingdom; ^6^Centre for Nutraceuticals, School of Life Sciences, University of Westminster, London, United Kingdom

**Keywords:** incentive in the workplace, prize, physical activity, employees health and wellbeing, team-based competition

## Abstract

**Introduction:**

The benefits of walking on health and well-being is well established and regarded as the most accessible form of physical activity (PA) that most individuals can incorporate into their lives. Despite the benefits, the impact of a competitive walking intervention combined with a prize incentive in the workplace is yet to be established. The aim of this intervention was to promote PA among university employees through teams-based competition with a prize incentive targeted towards the recommended 10,000 steps per day.

**Methods:**

A total of 49 employees participated and formed eight departmental teams ranging from Senior Admin management, Educational & Social work, Nursing & Midwifery, Sport & Exercise, Health Sciences, Admin Assistant, Library, and IT to compete in a walking intervention. Each team was handed an ActiGraph wGT3X-BT from Monday to Friday to record their walking steps. Steps. Post intervention participants completed an open-ended survey to provide their views about the intervention.

**Results:**

The ActiGraph findings determined that steps increased by 4,799 per day from daily baseline of 5,959 to 10,758 throughout this intervention. The themes from qualitative data showed that the prize incentive and competitive nature of this intervention has motivated staff to walk more, changed their behaviour, enjoyed the team-based competition, and improved perceived productivity in the workplace.

**Discussion and conclusion:**

This intervention increased employees’ daily steps by 4,799 and met the 10,000 steps guideline. The ‘Health Sciences’ team recorded the highest steps 531,342 followed by the ‘Education and Social Work’ accumulating 498,045 steps throughout this intervention. This intervention with prize incentive demonstrated a positive impact on employees personal and work-based outcomes as well as contributed to the workplace PA, health, and wellbeing literature, and more specifically, to the scarce research focused on university settings.

## Introduction

Walking has generally been acknowledged as a convenient and free form of exercise that can be integrated into everyday life (1, 2). The benefits of walking are well-established and include reducing the risk of cardiovascular diseases, diabetes, obesity, and depression (3–5). The physical activity (PA) guidelines are established to encourage individuals to engage in regular PA behaviour. Walking is the most accessible form of PA that most individuals can incorporate into their lives (6, 7). The walking guidelines differ regarding the recommended number of steps. For instance, Patel et al. suggested that 70,00 steps per day (8), whereas Wattanapisit and Thaname, recommended 10,000 steps (9). However, steps less than 5,000 are recognised as sedentary (10), while steps between 5,000–7,499 are identified as low active (11). Moreover, steps from 7,500 to 9,999 would be regarded as somewhat active, 10,000 steps are generally classified as active, and 12,500 or more per day is considered as highly active (9, 12). Nonetheless, 10,000 steps per day is generally accepted guideline worldwide (9, 13, 14). A range of workplace walking programmes reported a mixture of findings about health, wellbeing, and methodological approaches (15–18). Despite walking interventions featuring in the workplace, the methods and approaches used are questionable as most of the studies have mainly applied subjective methods for measuring steps (19, 20). However, studies have used more objective measures of accelerometers showed significant effects in step counts for the intervention group compared to the control group (*p* < 0.08) (21–23). Chan et al. recruited participants from five sedentary workplaces and delivered intervention to determine if accelerometer-based intervention increases daily steps instead of the self-reported methods (21). The results revealed average steps increased from 7,029 ± 3,100 to a plateau of 10,480 ± 3,224 and reported a significant decrease in body mass index (BMI), waist circumference, and resting heart rate.

Hallam, Bilsborough and Courten, conducted the 100-day 10,000 step workplace challenge and the results showed small but significant positive effect in symptoms of depression, anxiety stress and wellbeing. The positive effect occurred regardless of participants reaching the 10,000-step goal (24). The study shows the importance of workplace step challenges for employee’s health and wellbeing. Macniven et al. conducted the Global Corporate Challenge and Step Count Challenge recruiting over 585 participants from university in Australia. The findings indicated; daily average steps increased from 11,638 steps in week 1 to 13,787 daily steps in week 16 (*p* < 0.001). Although, this intervention had small to non-significant outcome on reducing the weight (−0.12 kg; *p* = 0.416), BMI x (−0.06 kg/m2; *p* = 0.314), and waist circumference (−0.43 cm; *p* = 0.082) but sitting time during work reduced significantly by 21 min per day (*p* < 0.001). However, in this study 92% of participants were meeting the 10,000 steps per day guideline at a baseline level leading to 98% at follow-up (25). This study concluded that such interventions are more attracted to female and younger employees including those who were already active (25). This shows there is a need to reach and target less active and hard to connect groups of employees in the workplace. Furthermore, Niven et al. conducted the Step Count Challenge and results showed reduction in stress and improved productivity (26). Although, the effectiveness of walking interventions is positive for increasing steps across various settings but more high-quality research is warranted in this area (27, 28).

Previous research has concluded the impact of walking combined with other activities or incentives are yet to be established (29, 30). For example, a 26 weeks intervention was designed to assess if financial incentive played a role in improving PA among hospital staff (31). The PA engagement was objectively measured, and intervention was tailored for individual and team-based, with results demonstrating that providing a financial incentive successfully increase the daily step counts (31). The importance of using financial incentives has increased across settings because research has demonstrated that extrinsic motivation is linked with PA participation. For instance, Patel et al. conducted a 13-week intervention to determine if a financial incentive increased team-based competitive step counts (8). The team with the most recorded steps was announced as a winner. The teams that achieved the daily recommended steps were awarded $50 (8). The findings revealed that competitive nature and financial incentives can help motivate teams to walk. However, the daily recommended steps in this study were set to 7,000. Other studies also recommended that offering extrinsic rewards influence social activities within teams that can improve walking among workers in different settings (8, 32).

Previous research also provided a general insight into the impact of step-counts, although most studies have only focused on financial incentives. Moreover, most of the previous research was grounded on the standard economic theory, which commonly accepts that people perform rationally (31, 33, 34). Previous research suggested that social and behavioural economic research design and implementing the incentives have an important influence (8, 35). Studies recommended that behaviour change interventions may be influential when people participate together, especially when socially connected, such as friends, family, or colleagues (8, 36, 37). Previous interventions have limitations such as the influence of team-based competitive intervention is yet to be explored (19). Some studies conducted walking interventions targeting university employees, and results found a significant effect between pre versus post intervention (*p* < 0.002) 23. Similarly, Fountaine et al. evaluated the differences between job roles in university staff, and results established the management staff accumulated significantly more steps than administrators and faculty staff (*p* < 0.05) (38). However, university employees did not reach the recommended number of daily steps. In the previous research, the actual steps taken and what is perceived as daily recommended steps were not recorded (32). Therefore, behaviour change and team-based competitive interventions assessing steps objectively, with prize incentive and exploring the influence qualitatively, may accomplish the gap regarding the walking intervention in the workplace (39). In summary, the existing research indicates that workplace step challenges can enhance PA and daily steps including positively impacting mental health and some work-related outcomes such as productivity. However, more research with stronger and comprehensive approach is needed. Thus, the aim of this intervention was to promote PA among university employees via a team-based competition with a prize for the winning team targeted towards the recommended 10,000 steps per day to present that steps-challenge intervention can reinforce the promise of workplace walking initiatives.

## Methods

### Participants

Following an institutional ethical approval, participants were recruited via an opportunistic sample using those who had participated in earlier studies of a broader piece of research from a UK higher education institution (university workplace) in Birmingham, based in West Midlands, England (40–42). All participants had to be adults (>18 years old), and currently employed by the participant university. A total of 49 employees participated in this mixed methods intervention and formed eight teams according to different job roles to compete. Previous research has reported that a single methodological approach is not ideal as the overarching research, may require the combination of methods such as mixed methods. For instance, PA levels can be assessed using quantitative approach while qualitative methods can explore participants of PA and team-based challenge. Therefore, a mixed-methods approach was adopted as it increases the strength and reduces weaknesses of paradigms in qualitative and quantitative research when used in isolation (43, 44). Table 1 provides a summary of the participant teams per job roles and the number of employees participated in each team.

**Table 1:** The breakdown of participants.

Teams per job roles	Number of staff participated in the intervention
Senior Admin management	4
Educational & Social work	7
Nursing & Midwifery	8
Sport & Exercise	3
Health Sciences	8
Admin Assistant	4
Library	5
IT	10

### Measures and protocols

The accelerometer used for steps recording was the ActiGraph wGT3X-BT, which is a valid and reliable monitor for measuring PA (40, 41). The ActiGraph can be positioned in various places on the body, such as hip, wrist, wrist, and ankle or even in the pocket. Device positions can affect the monitoring’s accuracy, which can impact the accuracy of the data collection (42). For instance, Hasson et al. assessed the validity of PA monitoring, and showed the data of participants who placed the monitor in the pockets were five times higher compared to monitors on hip (45). Due to the varying results, there is no generally accepted position or standardisation for wearing an ActiGraph. In this study, participants were informed that the monitor has to be worn on the wrist like a watch. Previous literature has supported the use of ActiGraph around the wrist because research have shown that participants are more likely remembering to wear the ActiGraph and the results against other body locations were more accurate representation of the steps (46–52). Moreover, due to the nature of the ActiGraph wGT3X-BT it remains sealed, as participants could not see or read their activities data recorded unless downloaded via a software which only the researcher had access to in this study. Therefore, for ActiGraph to function, an ActiLife, software version 6.13.3 was required for initialisation and downloading the data. Previous research has conducted to assess the effectiveness of using the wearable activity trackers-based (WAT) interventions supported in behaviour change techniques (BCT) in improving PA levels and reported positive findings regarding promoting PA levels (53, 54). However, Liu et al. concluded that WAT and pedometers could results in increasing PA levels but over a short period of time (55). The steps data in this study were recorded for 1 week as a baseline where participants were informed to wear the ActiGraph and conduct their typical activities as usual. Subsequently the intervention was implemented for 7 weeks and the data for each week was collected. Post intervention, teams were sent a qualitative open-ended survey to complete to understand their experience and perspective about the impact of this intervention.

For a department to be eligible to participate, a minimum of three individuals were required to compete. If there were more than three people in one team who wished to participate, they had the option to share the participation across the intervention period. For instance, one could decide what specific week to compete in the 10,000 steps challenge. The procedure was that each team had to nominate a leader responsible for collecting and returning the ActiGraph weekly. The researcher would visit every office and hand out the ActiGraph at 8:30 am, and the ActiGraph would start recording the data at 9:00 am on Monday till 17:00 on Friday. This was to represent a typical working day/week. The ActiGraph would then be collected after 17:00 on a Friday from offices. Teams were to compete against each other using a generic league format across the 7 weeks, with each ‘match’ accumulating the total steps from teams to form respective results. Table 2 provides the breakdown of weekly fixtures for the 7 weeks for each team.

**Table 2:** The breakdown of teams and fixture during the 7-week steps challenge.

	Sport & exercise	*vs*	Academic services management
Week 1	Library	*vs*	IT
	Health Sciences	*vs*	Nursing & Midwifery
	Education & Social Work	*vs*	Admin assistants
	Sport & Exercise	*vs*	Library
Week 2	Academic Services Management	*vs*	IT
	Admin Assistants	*vs*	Health Sciences
	Nursing & Midwifery	*vs*	Education & Social Work
	Sport & Exercise	*vs*	IT
Week 3	Academic Services Management	*vs*	Nursing & Midwifery
	Library	*vs*	Admin Assistants
	Education & Social Work	*vs*	Health Sciences
	Sport & Exercise	*vs*	Admin Assistants
Week 4	Academic Services Management	*vs*	Health Sciences
	Library	*vs*	Education & Social Work
	IT	*vs*	Nursing & Midwifery
	Sport & Exercise	*vs*	Nursing & Midwifery
Week 5	Academic Services Management	*vs*	Education & Social Work
	Library	*vs*	Health Sciences
	IT	*vs*	Admin Assistants
	Sport & Exercise	*vs*	Education & Social Work
Week 6	Academic Services Management	*vs*	Admin Assistants
	Library	*vs*	Nursing & Midwifery
	IT	*vs*	Health Sciences
	Sport & Exercise	*vs*	Health Sciences
Week 7	Academic Services Management	*vs*	Library
	IT	*vs*	Education & Social Work
	Admin Assistants	*vs*	Nursing & Midwifery

Teams were awarded 1-point for each day they as a team accumulated 10,000 steps. The team who accumulated the most steps in the week were awarded an additional 3-points for winning the ‘match’ in that specific week. Participants were aware that the team had an award with the most steps taken at the end of the intervention (i.e., winner of the league). There was also a weekly update on the league, updating the daily and total steps and points accumulated, showing teams winning on that particular week as a form of incentive as previous research suggested that social comparison through leaderboards or similar processes can help promote PA levels and competitiveness among teams (56–58). The league update was emailed to the leader of every team weekly and then the team leader would share the results with rest of the team. Research also reported that behaviour change interventions may be more influential when people participate together, especially when socially connected, such as friends, family, or colleagues (8, 36, 37). Providing a weekly update about the accumulated steps was to ensure participants were aware of how many steps they had taken each day and on that specific week and to plan accordingly for the next competition in subsequent weeks during intervention period. Table 2 provide the breakdown of weekly fixtures for the 7 weeks intervention for each team.

### Statistical and thematic analysis

Before the data analysis, teams were categorised according to their departmental names. The descriptive statistics of ActiGraph data is analysed as total baseline and intervention daily steps. Additionally, qualitative data was analysed via thematic analysis (TA) and presented as themes, sub-themes, and examples from the raw data. The TA is an approach that identifies, organises, allows interpretation, and reporting themes that are instigated from the set of data (53, 54). Furthermore, TA provide the impact of a given activity from participants’ perspective (59). Therefore, TA was conducted, and the six-step framework was considered an appropriate approach for qualitative data analysis for this intervention (54). Previous research supported and recommended using the six-step framework and regarded it as an effective approach when analysing data, as it provides a structure for conducting analyses through stages (59). This intervention also used the commonly applied trustworthiness model, which is regarded as the most fitting for the research purpose (53, 56). For instance, the model of trustworthiness for the results, consisting of five conditions: credibility, dependability, conformability, transferability, and authenticity, combined to construct trustworthiness (60). Furthermore, all data was member checked from the raw data through to complete analysis for data saturation.

## Results

**Figure 1:**
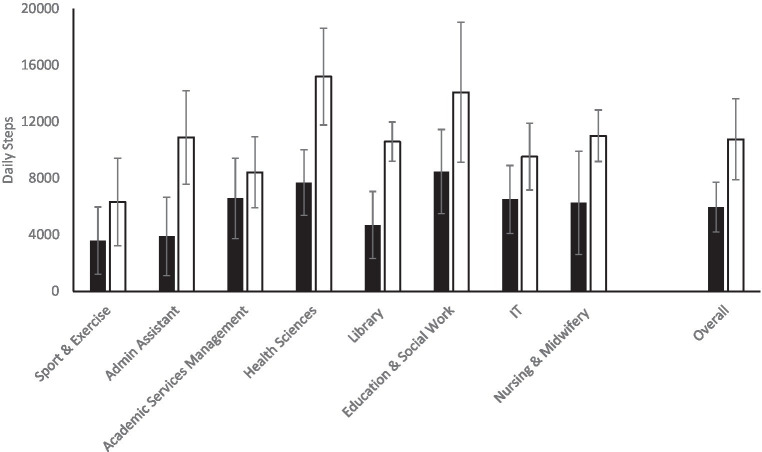
The mean and standard deviation (SD) of daily steps across the different participant groups and overall. Black bars = baseline; white bars = intervention.

Figure 1 shows the overall differences between baseline and intervention daily steps, and the differences between the baseline and intervention steps per each participant team according to the job roles. Table 3 highlights, themes, sub-themes, and examples from the raw data with the number of teams.

**Table 3:** Employees perspectives about participating in the team-based steps challenge intervention.

Themes	Sub-themes	Selected quotes from employees	Number of teams
	Team	*“We as a team never go outside at lunchtime but with the challenge, we have tried to do this every day. It motivated us as a whole team to walk more.*	8
Motivation	Encouragement	*“It encouraged us to take longer routes rather than quickest.*	7
	Stairs	*“We were motivated and started to take the stairs instead of the lift.*	4
Competition	Challenge	*“The whole team was competitive, and everybody just wanted to win, and we kept walking more.”*	8
	Healthy	*“Nice to be involved in such a healthy and shared competition”*	4
	Interesting	*“It was an interesting experience as we were never involved in such an activity before.”*	3
	Enjoyable	*“We felt excited, and it was fun to get involved in a workplace challenge and compete with other departments”*	7
Enjoyment	Fun	*“It was great fun partaking in this competition, absolutely loved it”*	3
	Excited	*“The whole team was very excited, and we kept talking about it all the time”*	3
Active	Fitness	*“We feel, it made us much fitter than before, and we started to take the stairs more often”*	6
	Walking	*“We got up and walk around more than we might have done otherwise”*	8
	Walking meetings	*“We were trying to take more walking meetings to record more steps and be active as we saw the positive effect”*	5
Productivity	Alert	*“I think the challenge helped improved our productivity and we felt alert all the way from the start to end of each week”*	5
	Productivity	*“It made us more productive because we would go for a walk as a team and still manage to get our work done on time”*	4
	Refreshed	*“This challenge helped us by regularly walking around the building or outside which helped kept us fresh throughout the day”*	5
	Breaks	*“The 10,000 steps challenge gave us the opportunity to take regular breaks which really helped us in a working day”*	7
	Sedentary behaviour	*“We have been conscious of sitting for a long time in the workplace. This intervention has changed our behaviour towards walking, now we look for an excuse to go for a walk in the workplace”*	8
Behaviour change	Consciousness	*“It made the team realise there are many benefits to walking and getting up within the working day and moving around is important. Getting fresh air throughout the day definitely helped. This challenge really changed us for better”*	5
	Communication	*“Improved our communication and we kept planning as to who wears the tracker and when and also, we were more inclined to speak to each other about non-work-related activity, something we never done before”*	6
	Gym	*“There has been noticeable difference in overall health and wellbeing during this period and the team felt this is necessary to continue and some colleagues have actually joined gym and started to run and walk more often, thanks to this intervention”*	2
Future incentives	Health and wellbeing	*“We would be keen in participating in more interventions like this within workplace, which would improve our health, and wellbeing”*	8
	Friendly	*“We will definitely participate again. It was an enjoyable challenge and was nice and friendly competition in the workplace”*	5
	Competition	*“It was an enjoyable experience, and everybody agreed they would partake in the same or something similar competitive programme in the future”*	8

The baseline steps data shows that employees were not meeting the 10,000 daily steps guideline and recorded an average of 5,959 steps per day as a baseline. The intervention data has shown that the average employees’ daily steps has increased to 10,758. Five participants teams shown to be meeting the recommended guideline of 10,000 steps per day, with the “Health Sciences” recording the highest daily average 15,193 steps per day followed by the Education and Social Work team who recorded 14,092 steps per day. The Sport and Exercise team recorded the least average steps per day (6318) followed by the Academic Service Management who recorded 8,430 steps per day.

With respect to the qualitative findings, a total of eight themes with several sub-themes were identified from the raw data providing an insight into employees’ perspectives about their participation in this intervention.

## Discussion

The baseline data demonstrated that participants were not meeting the 10,000 daily steps guideline. The findings of this intervention also suggests that this intervention increased the step counts towards the recommended daily allocation, such as increasing daily steps by 47,999 from baseline of 5,959 to 10,758. The improvement could be due to the nature of this intervention being a team-based competition with weekly incentives to compete and a prize for the winning team at the end of the intervention. It is also possible that the ActiGraph itself may have served as a reminder for participants to be more active. This may have changed employees’ behaviour and motivated them to go for a walk, visit colleagues rather than emailing/phoning or take stairs instead of lifts in the workplace. The present findings support previous research, suggesting team-based competitions with extrinsic rewards increase daily step counts (31, 32).

Overall, employees accumulating over 10,000 steps daily in this intervention. Though, discovering the differences between the baseline data and intervention among teams was important to identify if this intervention has improved daily steps between job roles and if any team have met the recommended steps guidelines as detailed in Figure 1. The number of steps appeared higher during this intervention than the baseline data as five departments were meeting the recommended steps guideline of 10,000 steps, with ‘Health Sciences’ recording the highest daily steps of 15,193. The increased number of steps among all teams could be due to the competitive nature of this intervention’s that staff may not wanted to lose against another team. The outcome of this intervention supports previous research suggesting that the competitive nature of intervention motivated staff not to give up and lose to other teams (8, 32). Another potential reason for step increment could be the prize incentive at the end intervention for the winning team. Our findings also align with previous research, suggesting that offering rewards can result in promoting PA participation and improves health (57, 58). Moreover, teams’ recording different steps could also be because of their job roles ranging from academics to technicians to management and administration. Indeed, some jobs may have been more physically demanding than others. For instance, the ‘Academic services management’ job requires staff to be sedentary, whereas the IT team requires staff to move around the building for IT-related issues. Despite the increase in daily steps, not all teams met the 10,000 steps guideline but making comparisons with baseline data shows a positive increase in daily steps. Participants from the winning teams were awarded £10 voucher each to ‘Mr. Mulligans’. They arranged a day to visit together for an indoor fun game and lunch as a team. The present findings support previous research suggesting, teams-based competition with an award incentive increased PA levels and improved health and well-being (31, 32).

With respect to the qualitative findings, a total of seven themes with several sub-themes were identified from the raw data providing an insight into employees’ perspectives about their participation in this intervention. All eight teams suggested this intervention had motivated them to walk more for different reasons, including enjoyment, health, winning, and perceived productivity. This shows that although some participants who were not reaching the 10,000 steps might still feel the benefits of participating in a competitive intervention, they may have led to increased step counts, relatedness to other team members, and enjoyment. Previous research determined that being autonomous, extrinsic, and intrinsic motivation can lead to increased exercise participation (61, 62). Therefore, introducing a team-based competitive intervention might be an ideal for promoting active behaviour in the workplace among employees. The extrinsic motives such as monetary prize at end of intervention for the winning team could be associated with participants motivation, competitiveness, and behaviour change towards PA engagement. For example, participants stated that; *“The whole team was competitive, and everybody just wanted to win, and we kept walking more.”* This supports previous research, concluding that extrinsic motives are linked with PA/exercise participation with favourable outcome (63, 64). Moreover, participants in this intervention stated that; *“We as a team never go outside at lunchtime, but with the challenge, we have tried to do this every day. It motivated us as a whole team to walk more.”* Employees never went outside during lunchtime, but this intervention motivated them to go outside as a team for a walk. Thus, positive effects on employees and changing behaviour from sedentary to active may have contributed to their health, wellbeing, perceived productivity, and served as an alternative strategy for teams to be active.

Previous literature mainly focused on participation, adherence and assessing variables in age, gender, culture, and tended that sport/PA competitive nature is typically for youth (45). Whereas, Bell et al. and Davey et al. noted, adults are likely to report competition as an essential factor for engaging in action (65, 66). Thus, this intervention provides a new concept of providing team-based competitive intervention that could improve PA levels, lead to enjoyment and perceived productivity and behaviour change among employees in the workplace. Additionally, this intervention positively contributed, changed behaviour from sedentary to active and raised consciousness about the importance of PA engagement and its impact on health and well-being among employees. Employees noticing the positive outcome during this intervention has led them to join gym membership, started walking and running to work, and continuing the active behaviour was considered an important. This supports previous research suggesting that change includes consciousness-raising, and this is regarded as one of the most important factors for behaviour change to occur (67). Though, changing behaviour is not simple, but making individuals’ aware of the pros, cons, and potential consequences of their actions on health, wellbeing and providing alternatives may help them contemplate as such was the case in this intervention as outlined by participants; *“It made the team realise there are many benefits to walking and getting up within the working day and moving around is important. Getting fresh air throughout the day definitely helped. This challenge really changed us for the better.”* After consciousness-raising, employees started to find alternatives for achieving more steps, such as conducting walking meetings and walking to colleagues’ desks rather than emailing, to take more steps than the team they were competing against. This shows that this intervention contributed to employees’ creative thinking and made them aware of the alternatives of meetings and being active rather than conducting usual meetings or emailing in a sedentary manner. Employees walking to colleagues’ rather than emailing could positively change the sedentary behavioural culture in the workplace as this may have encouraged them to walk more than sitting for a prolonged time. Employees being motivated and competitive throughout this intervention has positively changed their behaviour from inactive to active. The present findings contributes to the Transtheoretical Model (TTM) (68), Self-Determination Theory (SDT) (69) and Social Ecological Model (SEM) (70). The findings of this interventions highlighted that employee’s behaviour changed from sedentary to active and reported improved health and wellbeing and took time out to go for a walk during lunchtime or conducting a walking meeting leading to connectedness and relatedness with colleagues and people’s behaviour is not merely influenced by intrapersonal characteristics but also by various social factors which evidenced in this intervention. Therefore, there is a need for future research to investigate the walking interventions in the workplace considering applying a combination of behaviour change theoretical framework such as TTM, SDT and SEM.

### Limitations and future directions

Future research could build upon the framework of this intervention and the current findings can be generalised to other settings. Future team-based friendly competitive activities research is needed across settings and among university employees. Although all teams did not meet the 10,000 steps guideline, this intervention’s recorded steps have improved between teams and demonstrated the positive impact of competitive team-based intervention with prize incentives in the workplace. Despite the present intervention revealing useful findings, it is not without limitations. One of the key limitations of this intervention were that it did not have a follow-up study to evaluate if employees continued walking as teams in lunch times, taking stairs instead of lifts or conducting walking meetings instead of usual seated meetings or to assess if their active behaviour has relapsed when the intervention ceased. However, the possible explanations for not conducting the follow-up study were due to the COVID-19 pandemic, and national lockdown in the United Kingdom as most staff, especially the university employees had to work from home during this period.

## Conclusion

In summary, this intervention has increased employees’ daily steps by 4,799. The ‘Health Sciences recorded the highest steps of 531,342 followed by the ‘Education and Social Work’ team accumulating 498,045 steps throughout this intervention. Despite improving step counts in all teams and sparking a positive atmosphere in the workplace, this intervention also motivated employees to continue engaging in walking and improving their perceived productivity and changing their PA behaviour. This intervention contributes to the existing workplace PA, health, and wellbeing literature and more specifically, to the scarce research focused on university employees. Walking is beneficial for physical and mental health, and the increased steps evident in this intervention may have positively contributed to employee’s health, and wellbeing. The present findings support previous research concluding that 10,000 steps challenge positively change behaviour and improve health and wellbeing in the workplace (24). Thus, employees recognising the benefits of walking during the working day may be the cause for the positive perceived influence on behaviour change. Despite motivation, competency, and behaviour change, employees suggested that they were willing to participate in the future workplace interventions if focused on PA, health, and wellbeing. Employees willingness to engage in a similar intervention in the future, increased steps, and overall positive experience testify the success and positive impact of this intervention. The participant university and extended working environments could adapt the approach of this intervention to plan same/similar interventions for promoting healthy and active lifestyle in the workplace for all employees in the future.

## Data availability statement

The datasets generated and/or analysed during the current study are not publicly available due [to PhD research] but are available from the lead author on reasonable request.

## Ethics statement

The studies involving humans were approved by Birmingham City University, Health, Education & Life Sciences Faculty Academic Ethics Committee. The studies were conducted in accordance with the local legislation and institutional requirements. The participants provided their written informed consent to participate in this study.

## Author contributions

AS: conceptualization, methodology, software, validation, formal analysis, investigation, resources, data curation, writing – original draft preparation, visualization, and project administration. AS, MGZ, SD, MC, AK, and NW: writing – review and editing. MC, AK, and NW: supervision. All authors contributed to the article and approved the submitted version.
